# 0989. Accuracy of delivered airway pressure during proportional assist ventilation +. A bench study

**DOI:** 10.1186/2197-425X-2-S1-P74

**Published:** 2014-09-26

**Authors:** F Beloncle, N Rittayamai, E Akoumianaki, L Brochard

**Affiliations:** Critical Care Department, St Michael's Hospital and Keenan Research Centre, Toronto, Canada; Département de Réanimation Médicale, CHU d'Angers, Université d'Angers, Angers, France; Faculty of Medicine Siriraj Hospital, Mahidol University, Bangkok, Thailand; Intensive Care Unit, Klinik für Herzchirurgie, Herzzentrum, Leipzig, Germany; Interdepartmental Division of Critical Care, University of Toronto, Toronto, Canada

## Introduction

Proportional assist ventilation (PAV+) is a partial ventilatory support mode delivering airway pressure (Paw) in proportion to patient effort, enhancing patient-ventilator interactions. The ventilator estimates muscular pressure by using the respiratory system equation of motion with the instantaneous volume (V) and flow (V´) and the automatically calculated compliance and resistance. The mode gains in popularity but the accuracy of the delivered Paw by PAV+ is unknown.

## Objectives

To assess the accuracy of PAV+ by comparing the delivered Paw by the ventilator (Paw_meas_) to the theoretical Paw as defined by the equation of motion (Paw_Th_) and to examine the factors influencing this accuracy.

## Methods

An active servo lung (ASL5000) was programmed to resemble 4 respiratory mechanics: normal (Compliance (C)=60mL/cmH_2_O, Resistance (R)=10cmH_2_O/L/sec), obstructive (C=60, R=20), restrictive (C=30, R=10), and mixed (C=30, R=20). A Puritan-Bennett 840 ventilator with PAV+ was used. PAV+ was tested varying gain (30 and 60%), inspiratory trigger (IT) (0.8, 5 and 15 L/min), muscular pressure (Pmus) (10 and 15 cmH_2_O), positive end-expiratory pressure (PEEP) (0 and 5 cmH_2_O), and respiratory rate (RR) (10 to 30/min) to simulate intrinsic PEEP (PEEPi). PEEPi was measured using the Pmus curve. Paw_Th_ was calculated as follows: Paw_Th_=[(V/C)+(R×V´)]×Gain + total PEEP.

The inspiratory time was defined from the start of Pmus to the end of inspiratory V´. We calculated the difference between the mean Paw_meas_ and the mean Paw_Th_ during inspiration and between Paw_meas_ and Paw_Th_ at 25, 50, 75 and 100% of the inspiratory time. The percentage of difference between Paw_meas_ and Paw_Th_ was calculated as follows: %Δ=(Paw_meas_-Paw_Th_)/Paw_Th_ × 100.

## Results

Irrespective of respiratory mechanics and gain, mean Paw_meas_ was lower than mean Paw_Th_, Table1.

**Table 1:** Table 1

Gain (%)	Mechanics	Mean Pawmeas (cm H2O)	Mean PawTh (cm H2O)	Difference mean Pawmeas - mean PawTh (cm H2O)	mean Δ% (%)
**30**	**Normal**	6.6	9.4	-2.8	-29.8
	**Obstructive**	8.4	10.8	-2.4	-22.2
	**Restrictive**	7.0	8.5	-1.5	-17.6
	**Mixed**	6.7	8.8	-2.1	-23.9
**60**	**Normal**	9.6	13.7	-4.2	-29.9
	**Obstructive**	9.5	13.6	-4.1	-30.1
	**Restrictive**	10.2	13.0	-2.8	-21.5
	**Mixed**	10.3	13.5	-3.2	-23.7
**All conditions**		8.5	11.4	-2.9	-25.4

**Figure 1:**
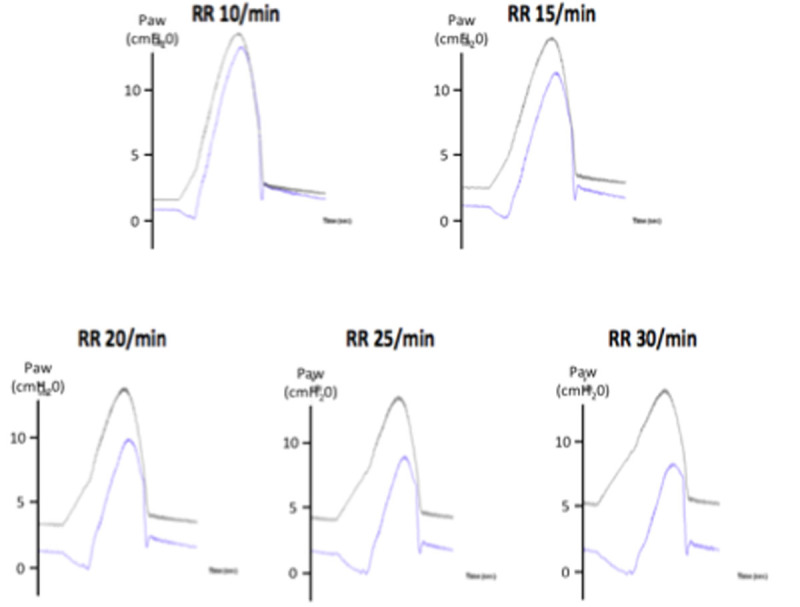
Representative tracings of measured airway pressure (Paw_meas_) and theoretical airway pressure (Paw_Th_) with progressive increases in rate (RR) and PEEPi. *Black lines:* Paw_Th_ wareforms. *Blue lines*: Paw_meas_ waveforms. Inspiratory trigger = 5 L.min; PEEP = 0 cmH_2_O; gain = 60%; Pmus = 10 cmH_2_O; Resistance = 20 cmH_2_O/L/s and compliance = 60 mL/cmH_2_O.

This underassistance by the ventilator was greatest at the beginning (25%) of the cycle and decreased later (75%) in inspiration. These findings were replicated under different IT, Pmus or PEEP settings. A high IT led to greater underassistance at the end of inspiration versus a low IT. A high Pmus was associated with a greater underassistance during the entire inspiration versus a low Pmus. A decrease in PEEP was associated with a major underassistance at the start of the inspiration. A higher RR resulted in a higher %Δ, showing that PEEPi increases total trigger delay and affects PAV+ accuracy, fig. 1. Combining the data from all conditions, PEEPi was correlated with the mean %Δ (R^2^=0.61, p< 0.001).

## Conclusions

PAV+ assistance is globally accurate compared to Paw_Th_ even if underassistance is often observed, especially at the start of inspiration. PEEPi leading to increased trigger delay is a major factor contributing to PAV+ inaccuracy. Clinical recommandations should include using a high trigger sensitivity and a careful PEEP titration when PEEPi is suspected.

The research laboratory has received grants from Covidien

